# Books and Magazines

**Published:** 1908-11

**Authors:** 


### Books and Magazines.

---

Electrical Treatment. By Wilfred Harris, M. D., F. R. C. P.; Physician to Out-Patients; Physician to Department of Nervous Diseases, and Lecturer on Neurology, St. Mary's Hospital, Maida Vale. Illustrated. 375 pages. Cloth. Price, $2.25, net. W. T. Keener & Co., Publishers, Chicago, 1908.

This is one of a series of publications on "Modern Methods of Treatment" and is intended to give a comprehensive view of the various forms of electrical treatment as practiced today, giving particular attention to work that may be done in medical practice with a good faradic and galvanic battery; for instance, the great relief that may be afforded in acute sciatica. Every progressive physician knows the value of electro-therapeutics, but few know the technique. This book will be a great enlightener.

---

Suggestive Therapeutics, Applied to Hypnotism, Psychic Science. By Henry S. Munro, M. D., Americus, Ga. Illustrated. C. V. Mosby, Medical Book & Publishing Co., St. Louis, 1907. Cloth and gilt. 350 pages. Price, $3.00.

This is a remarkable book. It is intensely interesting and very instructive. It looks to me like a faint light breaking in upon the darkness and mystery that surround what it is common to call "psychic phenomena," and upon the undoubted cures that are effected by faith healers and Christian Science. We are but in the A B C of the influence of the mind upon the body. Phenomena are observed and recorded, which look "miraculous." But there are no miracles; the laws that govern these phenomena are not yet understood, and certain ones that in all seriousness are attributed to "spirits" by such wise men, for instance, as Sir Oliver Lodge are, in the present state of knowledge, inexplicable. Evolution will bring to light these laws; man will learn to interpret them, and my prediction is that when understood all such phenomena will fall under the laws that govern molecular mechanics. Thought and all mental action is a force, and is "material"; it is the analogy of electricity, if not identical with it—whatever electricity is, for no one knows anything of it except by its manifestations. Buy this book.

---

EXAMINATION OF THE EAR.—A Practical Guide to. By Selden Spencer, A. B., M. D., Instructor of Otology in Washington University, St. Louis, etc. With an introductory chapter by H. N. Spencer, M. D., LL. D., Professor of Otology in Washington University. Cloth. 65 pages. Price, $1.00 C. V. Mosby, Publisher, St. Louis, 1908.

The object and use of this manual are sufficiently indicated in the title. The illustrations, showing the anatomy of the ear, are a great help to a clear understanding of pathological conditions of that organ.

---

COSMETIC SURGERY.—The Correction of Featural Imperfections. By Charles C. Miller, M. D. Second edition enlarged. Including the description of numerous operations for improving the appearance of the face. 160 pages. 96 illustrations. Prepaid, $1.50. Published by the author, 70 State Street, Chicago.

---

THE CURE OF RUPTURE BY PARAFFIN INJECTIONS. By Charles C. Miller, M. D. Comprising a description of a method of treatment destined to occupy an important place as a cure for rupture owing to the extreme simplicity of the technic and its advantages from an economic standpoint. Published by the author, 70 State Street, Chicago. Prepaid, $1.00.

---

GOLDEN RULES OF DIETETICS (Medical Guide and Monograph Series.)—The General Principles and Empiric Knowledge of Human Nutrition; Analytic Tables of Foodstuffs; Diet Lists and Rules for Infant Feeding and for Feeding in Various Diseases. By A. L. Benedict, A. M., M. D., Buffalo. C. V. Mosby, Publisher, St. Louis, 1908. Cloth. 390 pages. Price, $3.00.

The "science" of feeding is almost entirely disregarded, yet it is of fundamental importance. Every family should read and heed this book.

Diagnosis by the Urine; or The Practical Examination of Urine With Special Reference to Diagnosis. By Allard Memminger, M. D., Professor of Chemistry and Hygiene and Clinical Professor of Urinary Diagnosis in the South Carolina Medical College, etc. Third edition, enlarged and revised, with 27 illustrations. Cloth. 100 pages. Price, $1.00. P. Blakiston's Son & Co., Philadelphia, 1908.

This neat little volume is dedicated to "students of medicine, and practitioners at large, in the hope that its simplicity of arrangement and of style will commend it to them and be the means of increasing their knowledge and diminishing their labor in this special department of science."

We commend the book to our readers. It will be a great assistance in certain obscure conditions.

---

Subcutaneous Hydrocarbon Protheses. By F. Strange Knolle, M. D., author of "The Recent Roentgen Discovery," "The X-Rays," "Medico-Surgical Radiography," etc. Illustrated. Pages, 144. Cloth, $2.50. The Grafton Press, Publishers, New York, 1908.

Cosmetic Surgery is getting to be quite a distinct art. Here is another and more pretentious work, telling how that certain lost parts of the human system may be restored and look well (prothesis). All old doctors will recall and laugh at the crude operations of our ancestors—as figured in DeWitt's Surgery, for instance, the Talliacotion operation, plastic surgery. Not only does this book describe and figure operations for the restoration of parts, but tells how to correct deformities and to get rid of wrinkles, etc. The book will be found well worth while, as well as the price.

---

Genito-Urinary Diseases and Syphilis. By Edgar G. Ballenger, M. D., Lecturer on Genito-Urinary Diseases, Syphilis and Urinary Analysis, Atlanta School of Medicine; Editor *Journal Record of Medicine;* G. U. Surgeon to Presbyterian Hospital, Atlanta, Ga. 86 illustrations. Atlanta, Ga.: E. W. Allen & Co., 1908. Cloth. 259 pages. Price not stated.

Dr. Ballenger has carefully and thoroughly scanned the latest authorities on these subjects as exemplified in the current medical publications and the large works, and has condensed the essence of their teachings in this little volume for the use, more especially, of medical students whose time must be economized. But it will also be a handy reference book for the practicer of medicine, whose time also must be husbanded. Dr. Ballenger has done his work well. No price is given with the copy sent me, but it is not expensive. I should say about a dollar.

Report From the Pathological Department of the Central Indiana Hospital for the Insane. Dr. Geo. F. Edenharter, Superintendent, Indianapolis, Ind. Wm. R. Burford, Contractor for State Printing, 1908.

This report is for the year 1903-'04-'05-'06. It gives a detailed account of the work done in the pathological department of the insane asylum—J. Allen Jackson, M. D., Pathologist; E. D. Martin, M. D., Assistant Pathologist. Dr. Edenharter has a staff of seven assistant physicians. At the Pathological Department (Annex), to which a beautiful building designed by Dr. Edenharter is devoted, a course of lectures is given by Professors Hutchins, Reger and Sterne of the Indiana University School of Medicine, and Dr. Jackson, the Pathologist of the asylum. Special clinics are provided for those who desire to take instructions in Mental Pathology. These will be conducted by Prof. E. H. Lindsay, of the University. Dr. Edenharter will be pleased to correspond with readers of the "Red Back."

---

General Surgery.—A Presentation of the Scientific Principles Upon Which the Practice of Modern Surgery is Based, by Ehrich Lexer, M. D., Professor of Surgery, University of Konigsburg; American Edition, edited by Arthur Dean Bevan, M. D., Professor and Head of the Department of Surgery, Rush Medical College, Chicago, Ill. An Authorized Translation of the Second German Edition by Dean Lewis, M. D., Assistant Professor of Surgery, Rush Medical College, Chicago, Ill. With 449 illustrations in the text, partly in color, and two colored plates. D. Appleton & Company, New York and London, 1908.

The appearance of this great work is epoch-making. It will create a stir in surgical circles, for it is something of a surprise. It is complete, and embraces the latest conceptions and accepted teachings. One may say that it is the net gains of the accumulated experiences of the surgical world; the kernel, winnowed from the chaff. In addition to this, much new matter has been added, which brings it up to now. Usually, new experiences, observations and discoveries float around in the journals and finally find lodging in the text-books a year or so later. It presents the present status of the science and art in a thorough, clean-cut, concise and practical way; and a wayfaring man, though a fool, need not err—after reading it. The addition to the text made by the editors and translators embrace the chapter on Blastomycosis by Dr. Oliver Ormsby. This disease is new to America where, alone, it has been studied. It is unknown, the translators say, in Europe; and also the chapter on Blood Examinations in Surgery by Dr.

Rosenow, and that on Opsonins and the Wright vaccination treatment; also an abstract of Dr. Geo. Crile's recent work on the direct transfusion of blood. These constitute a part of the new matter, while a number of the illustrations are taken from the clinics of Drs. Bevan and Lewis. Asepsis and Antisepsis are dealt with clearly and exhaustively; and the technic is given with each operation. Indeed, this splendid book—worthy of the great house of the Appletons—is a treasure-trove, which we cordially commend to the profession, and which we feel assured will be received by the better element with glad acclaim. DR. DANIEL.

---

THE PRACTICAL MEDICINE SERIES.—Comprising Ten Volumes on the Year's Progress in Medicine and Surgery. Under the general editorial charge of Gustavus P. Head, M. D., Professor of Laryngology and Rhinology, Chicago Post-Graduate Medical School.

We have received two volumes of 1908 series.

VOLUME I.—GENERAL MEDICINE. Edited by Frank Billings, M. S., M. D., Head of the Medical Department and Dean of the Faculty of Rush Medical College, Chicago, and J. H. Salisbury, A. M., M. D., Professor of Medicine, Chicago Clinical School.

VOLUME II.—GENERAL SURGERY.—Edited by Jno. B. Murphy, A. M., M. D., LL. D., Professor of Surgery in Rush Medical College (in affiliation with University of Chicago), Chicago. The Year-Book Publishers, 40 Dearborn Street. Price, for the series of ten volumes, $10. Separately, Vol. I, $1.50; Vol. 2, $2.00.

---

DISEASES OF THE SKIN. By A. M. Ohmann-Dumesnil, A. M. M. E., M. D., Ph. D., etc., formerly Professor of Dermatology and Syphilology in the St. Louis College for Medical Practitioners; the St. Louis College of Physicians and Surgeons; the Marion-Sims College of Medicine; Member of the St. Louis Medical Society, of the Missouri State Medical Association, of the First, Second, Third, Fourth, Fifth and Sixth International Dermatological Congresses, etc. Third edition, thoroughly revised and enlarged. 140 illustrations. C. V. Mosby Medical Book & Pub. Co., Publishers, St. Louis, Mo. Price not stated.

Dr. Dumesnil is an expert in diagnosis in diseases of the skin. That is the main thing in practice, not alone here but in everything. Given the diagnosis, any good book will tell you what to do. Dr. D. lays stress on the importance of diagnosis. Like Mrs. Hale in her cook book, on How to Cook a Hare; first, get your hare. It is a clean, practical work on the subject, intended for the every-day general practicer of medicine, and is written in a pleasing style, in plain language from truthful James. This is the latest. Get it. You will be glad I put you next, at this early date. Just out; latest treatment. Finely illustrated with cuts from the author's own clinics. DR. D.

